# The efficacy of masks for influenza-like illness in the community: A protocol for systematic review and meta-analysis

**DOI:** 10.1097/MD.0000000000020525

**Published:** 2020-06-05

**Authors:** Hua Wang, Mao-Bing Chen, Wei-Yan Cui, Hua-Lan Xu, Qi-Han Zheng

**Affiliations:** a Department of ICU; b Department of Emergency, Wujin People's Hospital Affiliated with Jiangsu University and Wujin Clinical College of Xuzhou Medical University, Changzhou, Jiangsu, PR China.

**Keywords:** COVID-19, human, influenza, masks, meta-analysis

## Abstract

**Background:**

During the COVID-19 period, there was a huge gap in the understanding of masks between east and west. At the same time, the mechanism of the mask and the effect after use, also appeared differences. The Objective of this Meta-analysis is to systematically evaluate the efficacy of masks for influenza in the community.

**Methods:**

The Web of Science, PubMed, The Cochrane Library, EMBASE and Clinical Trials will be electronically searched to collect randomized controlled trials regarding the efficacy of masks for influenza in the community through Apr 2020. Two researchers independently screened and evaluated the obtained studies and extracted the outcome indexes. Revman 5.3 software will be used for the meta-analysis.

**Results:**

The outbreak is continuing, and we need to be prepared for a long fight. If masks are effective, we need to promote their use as soon as possible. If masks are ineffective, strong evidence should be given. This is an urgent task and our team will finish it as soon as possible.

**Conclusion:**

Provide stronger evidence to solve the problem, should we wear masks or not right now.

## 1 Introduction

Masks have been a controversial topic in the COVID-19 epidemic.^[1,2]^ In Europe and North America, most people believe that wearing masks is ineffective. In fact, most trials support this.^[3,4]^ In Asia, masks are considered a necessity, especially during outbreaks. Although there is no strong evidence of evidence-based medicine, outbreaks in Southeast Asian countries do tend to improve.^[5,6]^ What role does the mask play in this COVID-19? Is it necessary for everyone to wear masks? This analysis tries to solve this problem.

The mask originated in Europe as a bird-beaked masks, it took more than a hundred years of evolution to become what it is today. At present, masks are worn in the muzzle to prevent the wearer's respiratory secretions from contaminating others or the environment.^[7]^ Common masks or surgical masks have limited effectiveness in preventing the lungs from harmful substances entering from the environment. These are the basic parameters of the mask that tell us. More evidence is needed as to whether masks could protect people in the community.^[1]^

This study will take a neutral position and look for an answer that would convince most people.

## 2 Methods

Regarding the efficacy of masks on influenza in the community, there are 2 main categories: one is to distribute masks to each participant in the group; the other is to distribute masks to patients with influenza and study contacts. The second type of trials was studied in this meta-analysis.

### 2.1 Design and registration

A meta-analysis will be conducted to evaluate the efficacy of masks in patients with influenza. This protocol has been registered on the international prospective register of systematic reviews (PROSPERO), registration number is CRD42020179358 (https://www.crd.york.ac.uk/PROSPERO). No ethical approval is required since this study used data that will be already in the public domain.^[8]^

### 2.2 Study selection

#### 2.2.1 Study type

The study type is randomized controlled trials (RCTs).

#### 2.2.2 Study object

**Figure 1:**
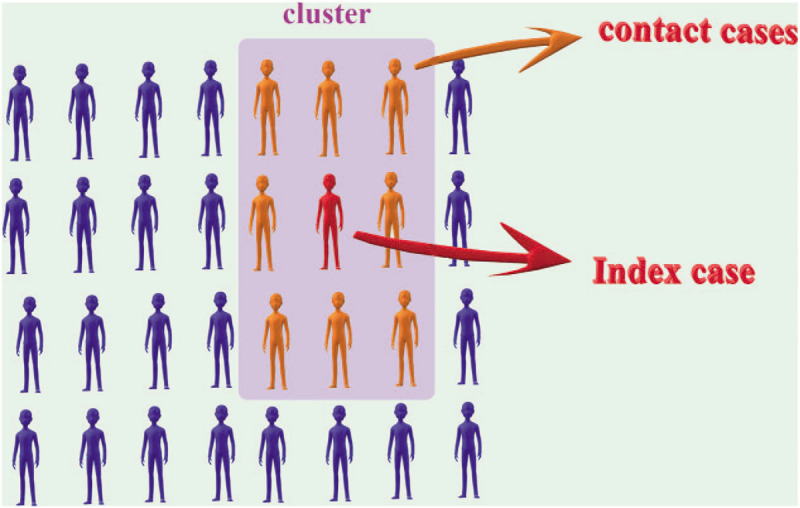
Schematic representation of cluster, index case and contact cases.

First we need an infected case called index case. The patient needed to have an influenza-like illness or a laboratory diagnosis of influenza. The people around his environment are called contact cases. These contact cases could not be enrolled with an influenza-like illness or influenza. We randomly divided these clusters into mask group and control group (Fig. 1).

#### 2.2.3 Intervening measure

In the Mask group, index cases should wear masks and live with contact cases as usual. There is no requirement for contact cases to wear masks. Index cases must report regularly.

In the Control group, index cases and contact cases live together as usually. There is no requirement for both index cases and contact cases to wear masks.

#### 2.2.4 Outcome indicator

The clinical diagnosis and laboratory diagnosis of influenza-like illness.

#### 2.2.5 Exclusion criteria

Studies with data that could not be extracted or utilized, studies with animal experiments; and literature reviews were excluded.

### 2.3 Data sources and searches

**Figure 2:**
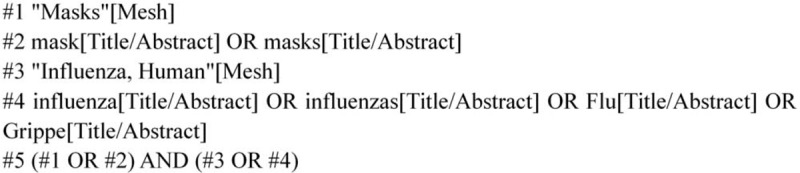
PubMed database retrieval strategy.

We will search English language publications through Apr 2020 using the following databases: Web of Science, PubMed, the Cochrane Library, EMBASE and Clinical Trials. The search terms included “masks” and “influenza”. In Figure 2, we use the PubMed database as an example.

### 2.4 Study screening, data extraction and risk assessment of bias

Data will be collected independently by two researchers. The unqualified studies will be eliminated, and the qualified ones will be selected after reading the title, abstract, and full text. Then, the research data will be extracted and checked, and disagreements will be discussed or a decision will be made by the authors. The extracted data include the following:

1. basic information of the study, including title, author and year of publication;
2. characteristics of the included study, consisting of the study duration, the sample size of the test group and the control group, and the intervention measures;
3. The outcome indicators and data; and
4. the information needed to assess the risk of bias.

The risk of bias in the included studies will be assessed using the RCT bias risk assessment tool recommended in the Cochrane Handbook for Systematic Reviews of Interventions (5.1.0). This work will also be done independently by two researchers.

### 2.5 Statistical analysis

The Revman 5.3 software will be used for this meta-analysis. The dichotomous variables will be expressed as the relative risk (RR) as an effect indicator and the estimated value and 95% confidence interval (CI) will be included as effect analysis statistics. The significance level sets at α = 0.05. A heterogeneity test will be conducted with the results of each study. If there is no statistical heterogeneity among the results (I^2^ ≤ 50%), meta-analysis will be performed by fixed effect model. If there is statistical heterogeneity among the results (I^2^ > 50%), the source of heterogeneity needs to be found and meta-analysis will be performed by random effects model. If we could not find the source of heterogeneity, descriptive analysis will be performed only.

### 2.6 Subgroup analysis

We will conduct subgroup analysis according to specific results. Examples include differences in trials methods, bacterial or viral infections, single infection or co-infection.

### 2.7 Assessment of publication bias

If more than 15 articles are available for quantitative analysis, we will generate funnel plots to assess publication bias. A symmetrical distribution of funnel plot data indicates that there is no publication bias, otherwise, we will analyze the possible cause and give reasonable interpretation for asymmetric funnel plot.^[9]^

### 2.8 Confidence in cumulative evidence

GRADE system will be used for assessing the quality of our evidence. According to the grading system, the level of evidence will be rated high, moderate, low and very low.^[10]^

## 3 Discussions

In the preliminary preparation, we found that many RCTs results do not support the effectiveness of masks in the community.^[11–13]^ But there seems to be a trend in the data that masks may have the potential to protect against influenza. Perhaps through meta-analysis, a positive result can be obtained from data synthesis. Of course, these should be built on the basis of seeking truth from facts.

At the same time, we also found other RCTs on masks. For example, masks are used in dormitory buildings^[14]^ or in hospitals.^[15]^ We believe that the difference between these RCTs and the included RCTs of our meta-analysis is large, and the heterogeneity is high, so these RCTs will not be included in this analysis for quantitative analysis. It can only be used as a reference for mask effect and a systematic review.

Maybe we cannot fight the epidemic alone, and we need a team. There is a lot we need to do, and masks may be a part of that. In the face of a disaster like COVID-19, even if the masks cannot be shown to be significantly effective, as an option, we could use it before the evidence is available. Just like a Chinese proverb, treating a dead horse tentatively as if it were still alive, which means that we should never give up for lost. We hope that with our efforts, the outbreak can be ended as soon as possible.

## Author contributions

Hua Wang and Mao-bing Chen proposed the concept of this study and designed this systematic review. Hua Wang registered the protocol of the systematic review and meta-analysis. Hua Wang, Qi-han Zheng, Wei-yan Cui and Mao-bing Chen were responsible for the collection, collation and statistical processing of the literature. All authors participated in the drafting of the first draft of the paper. Hua Wang reviewed and proofread the paper. All authors agree to publish the paper publicly.

**Conceptualization:** Hua Wang and Mao-bing Chen.

**Data curation:** Hua Wang, Qi-han Zheng, Wei-yan Cui and Mao-bing Chen.

**Methodology:** Hua Wang and Wei-yan Cui.

**Software:** Hua Wang and Hua-lan Xu.

**Supervision:** Hua Wang and Qi-han Zheng.

**Writing – original draft:** Hua Wang, Mao-bing Chen, Qi-han Zheng, Hua-lan Xu and Wei-yan Cui.

**Writing – review & editing:** Hua Wang.
